# Fitting dynamic models with forcing functions: Application to continuous glucose monitoring in insulin therapy

**DOI:** 10.1002/sim.4254

**Published:** 2011-05-18

**Authors:** DJ Lunn, C Wei, R Hovorka

**Affiliations:** a Medical Research Council Biostatistics Unit, Institute of Public Health, University Forvie SiteRobinson Way, Cambridge CB2 0SR, U.K.; b Diabetes Modelling Group, Department of Paediatrics, University of CambridgeCambridge, U.K.

**Keywords:** dynamic models, glucose kinetics, artificial pancreas, autoregressive models, WinBUGS

## Abstract

The artificial pancreas is an emerging technology to treat type 1 diabetes (T1D). It has the potential to revolutionize diabetes care and improve quality of life. The system requires extensive testing, however, to ensure that it is both effective and safe. Clinical studies are resource demanding and so a principle aim is to develop an *in silico* population of subjects with T1D on which to conduct pre-clinical testing. This paper aims to reliably characterize the relationship between blood glucose and glucose measured by subcutaneous sensor as a major step towards this goal. Blood-and sensor-glucose are related through a dynamic model, specified in terms of differential equations. Such models can present special challenges for statistical inference, however. In this paper we make use of the BUGS software, which can accommodate a limited class of dynamic models, and it is in this context that we discuss such challenges. For example, we show how dynamic models involving forcing functions can be accommodated. To account for fluctuations away from the dynamic model that are apparent in the observed data, we assume an autoregressive structure for the residual error model. This leads to some identifiability issues but gives very good predictions of virtual data. Our approach is pragmatic and we propose a method to mitigate the consequences of such identifiability issues. Copyright © 2011 John Wiley & Sons, Ltd.

## 1. Introduction

Type 1 diabetes (T1D) is a chronic autoimmune disorder characterized by dysregulated blood-glucose (BG) levels due to an inability of the pancreas to produce insulin, the hormone that promotes uptake of glucose by cells 1. Persistent exposure to high glucose levels (hyperglycaemia) causes long-term diabetes complications and organ dysfunction 2. The standard therapy is based on multiple insulin injections, using a combination of short and long acting insulin analogues, informed by frequent BG self-monitoring 3. Treatment by continuous subcutaneous insulin infusion (CSII 4) is on the rise and uses a portable electromechanical pump to mimic nondiabetic insulin delivery, infusing insulin at preselected rates—basically a slow basal rate with patient-activated boosts at mealtimes. However, intensive insulin therapy aiming to achieve near-normal glucose control is associated with an increased risk of low BG levels (hypoglycaemia), potentially leading to seizures, unconsciousness, brain damage and even death 5. Optimization of insulin therapy is confounded by large day-to-day and diurnal variability in insulin requirements influenced by factors such as exercise, stress, and recurrent illness 6–8.

Self-monitoring of BG offers only a snapshot, each time, of the underlying glucose excursion, thus, making for considerable uncertainty in determining the right treatment decision to achieve and maintain desirable glucose levels. Continuous glucose monitoring (CGM 9) devices offer an alternative approach informing on real-time glucose levels, with the possibility of real-time hyperglycaemia and hypoglycaemia alerts 10. CGM can show the rate at which glucose is increasing or decreasing, and thus facilitate an understanding of how glucose levels react to insulin, food, exercise, and other factors, thus, providing scope for finer glucose control.CGM devices use a sensor to measure *interstitial* glucose, which provides an indirect reading of BG. That they can lead to improved glycaemic control has been demonstrated 11. They may also reduce the frequency of hypoglycaemia events in people with well controlled T1D 12. CGM devices and insulin pumps can be combined to form an artificial pancreas. Insulin delivery is then automatically modulated according to real-time sensor-glucose (SG), as directed by a control algorithm, rather than at preselected rates as during the conventional ‘open-loop’ CSII 13.

An impediment to real-time accurate CGM tracing is the existence of a physiological delay between BG and interstitial glucose^14^–^16^. While there is great potential for CGM systems and the artificial pancreas to revolutionize diabetes care and improve quality of life, a more detailed quantitative understanding is needed about the relationship between BG and SG as reported by CGM devices. This information is helpful to health-care professionals, subjects with T1D and their carers, and also to facilitate *in silico* testing of the artificial pancreas 17. With this latter objective in mind, it is important to be able to predict *realistic* sensor data, since sensor data are what the control algorithm will have to respond to. Our approach is to characterize the relationship between BG and SG throughout the population using data from a relatively small clinical study. We use a nonlinear regression function to describe each individual's data and simultaneously estimate the population distribution of the underlying parameters, exploring both inter-and intra-individual variability, including any correlations between the parameters. We also use an autoregressive (AR) process to accurately describe the residual errors. The estimated population distribution can then be used to simulate realistic parameter sets for new individuals, with appropriate correlations between parameters. These can then be used, in combination with real or simulated BG data, to predict SG profiles for the new individuals, which are overlaid with simulated AR processes (informed by the estimated model) to account for typical differences between sensor observations and our regression function. A wealth of virtual data can thus be generated, allowing extensive testing and accelerated development of the artificial pancreas 17.

Existing CGM devices lack the accuracy of BG meters. Early reports documented particular concerns at low BG values 18, 19, although more recent assessments indicate comparable relative accuracy at normal and low glucose ranges 20, 21. Each new generation of CGM devices brings about improvements in accuracy, reliability, and sensitivity and specificity of hypo-and hyperglycaemia alerts 10, 21, 22. This has a positive effect on the utility and frequency of CGM use, which is associated with health benefits 23 and facilitates the development of the artificial pancreas 24, 25. Our work complements these developments by providing a methodological framework and insights into the nature and statistical properties of sensor errors. This may also inform the development of advanced control algorithms for the artificial pancreas 26.

The present paper is concerned with the Guardian® RT CGM system^27^. Breton and Kovatchev 28 use similar ideas to model another CGM system (FreeStyle Navigator™, Abbott Diabetes Care, Alameda, California). However, their estimation strategy is somewhat fragmented, with parameter uncertainty being ignored between the various stages. We extend their approach by combining all of the various modelling components into a single model, allowing all sources of uncertainty to propagate through to our final inferences. We also examine the process of calibrating the sensor in more detail, allowing the model to be extended to handle multiple calibration events for a single individual. Finally, we simultaneously model the inter-and intra-individual variability of system parameters in order to facilitate prediction.

The structure of the paper is as follows. In Section 2 we describe the data obtained from a small clinical study involving 12 children and adolescents. Section 3 provides some mathematical background and then presents the various aspects of our statistical model, including the dynamic sub-model and how we account for system calibration. Section 4 presents the results of our analyses, and a concluding discussion is given in Section 5.

## 2. Data

A total of *N* = 12 children and adolescents with T1D treated by continuous subcutaneous insulin infusion participated in a clinical research study conducted at the Wellcome Trust Clinical Research Facility, Addenbrooke's Hospital, University of Cambridge, UK 24. The sample size was not based on any power calculation as the study concerned (APCam01 in 24) was exploratory. Participants and, as appropriate, their carers gave informed consent/assent. The study was approved by the Cambridge Research Ethics Committee (REC Ref 06/Q0108/350).

A glucose sensor was fitted to each participant at least 24 h prior to the study, and, following a run-in period and calibration as suggested by the manufacturer, the Guardian® RT CGM system took SG readings every 5 min. BG was measured every 15 min by collecting samples via a venous cannula. The study ran from 17:00 until 12:00 the following day, giving *m* = 77 BG and *n* = 228 SG measurements for each individual. There are a small number of missing SG measurements, which we shall treat as unknown parameters in order to retain a balanced data set. Two self-selected meals were eaten at 18:00 and 08:00 the following morning to maintain a normal carbohydrate intake, each meal containing a mean (SD) of 87 (23) g carbohydrates. Prandial insulin boluses were given with the meals. During the study, the Guardian® RT was calibrated, using recent BG measurements, shortly after 17:00 and every 6 h thereafter, thus splitting the study period into five distinct ‘calibration periods’ for each individual.

## 3. Methods

### 3.1. Background

The model that we will describe in subsequent sections is essentially an extension of the non-linear regression


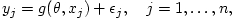


where *y*_*j*_ and *x*_*j*_ denote response and independent variables, respectively, θ denotes a set of regression parameters, and ɛ_*j*_∼N(0, σ^2^), say. As a simple example, let us consider a situation in which the regression function *g*() represents exponential decay and *x* is elapsed time:


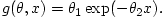
(1)

Note that (1) is the *unique* solution to


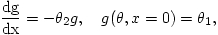
(2)

and so (1) and (2) are equivalent specifications of the same regression function. Now suppose that we can only express our regression function in terms of differential equations (with the corresponding initial conditions) as we do not know the analytic solution. If we know that a unique solution exists, however, then we know that solution is simply a deterministic function of the inputs, θ and *x*, albeit of unknown form. If we can find a way to evaluate the solution, we may thus treat it as we would any other deterministic function. We may then exploit standard graphical modelling theory 29, 30 to evaluate the full conditional distributions of any unknown inputs, e.g. θ_2_. In this paper, we make use of the BUGS software 31, 32 with *WBDiff* interface 33 installed (to allow specification of differential equations). The differential equations, described in the following subsection, are solved numerically by the software using a Runge–Kutta algorithm 34, and Metropolis–Hastings samplers 35, 36 are typically used for sampling the unknown inputs.

Now suppose that the differential equations depend on some additional quantity, such as the ambient temperature or pressure, say, whose evolution through time is driven by external factors. This happens in many settings. For example, wind stress may be a factor in modelling ocean circulation 37, whereas light intensity, temperature, availability of food/nutrients, and wind speed may all be important in ecological modelling 38. In our case, the equations depend on BG concentrations but in other areas of diabetes research insulin concentrations may be used, e.g. 39. We cannot usually model such quantities but may be able to observe their values over a series of times. If we interpolate between the observations, then we can approximate the relevant quantity at any time within the observation period. If this observation period envelopes the time-frame over which we wish to evaluate our regression function, then solving the differential equations is still possible, and the interpolated series is referred to as a *forcing function*. WBDiff has not been designed with the specification of forcing functions in mind, but we show how they may be accommodated in Section 3.5.

As we shall see later, our regression function (also referred to herein as the ‘dynamic model’) is somewhat imperfect—there are clear fluctuations away from the fitted model apparent in the observed data. For the purposes of testing the artificial pancreas' control algorithm, it is important that we are able to predict realistic sensor data, and so we consider an AR model for the residual errors (ɛ_*j*_ above)—see Section 3.3. We fit the AR and dynamic models simultaneously, to fully account for uncertainty and also to capture posterior correlation between them. However, we find that they are somewhat confounded and identifiability issues arise unless the degree of autocorrelation in the AR process is constrained. Our approach to this is pragmatic and involves exploring various ways of limiting the extent to which the two models may interact, as discussed at the end of Section 4 and in the discussion.

### 3.2. Glucose kinetics

The CGM sensor measures glucose concentrations in the interstitial fluid, which can be related, mathematically, to the BG concentration via a compartmental model 40, 41:


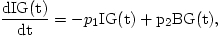
(3)

where IG denotes interstitial glucose. Hence, IG increases at a rate proportional to BG but is ‘used up’ according to a first-order process—the more there is, the faster it disappears.

The sensor does not measure IG directly but, instead, measures electric current in the interstitial fluid and maps this to a *scaled* measure of IG via an assumption of proportionality. When the system is calibrated, the appropriate scale is chosen by equating scaled current with recent measures of BG. To account for this calibration we transform (3) to the same scale, by defining *normalized* interstitial glucose NIG = νIG and choosing ν = *p*_1_/*p*_2_ so that NIG is equal to BG at steady state:


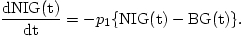
(4)

Let SG_*ij*_ denote the *j*th measured SG concentration for individual *i*(*i* = 1, …, *N* = 12, *j* = 1, …, *n* = 228). Similarly, let BG_*il*_, l = 1, …, m = 77, denote the *l*th measured BG concentration for individual *i*. Further, denote the times at which SG_*ij*_ and BG_*il*_ were measured by *t*_*ij*_ and *s*_*il*_, respectively. A simple model for fitting individual *i*'s data is then


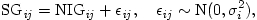
(5)

where NIG_*ij*_ is the solution to (4) at time *t*_*ij*_. This is a deterministic function of three unknown inputs: (i) the value of *p*_1_; (ii) the initial condition NIG(*t* = 0); and (iii) the form of BG(t). We assume that each individual has a distinct, but unknown, value of *p*_1_, which we denote by *p*_1_*i*__. Often the initial conditions will be known, but in general they are not, and so these may also be treated as unknown parameters; in this case denoted by NIG_0_*i*__, *i* = 1, …, *N*. Regarding the form of BG(t), we assume that linearly interpolating‡ between the observed values for each individual, BG_*il*_, l = 1, …, m, provides a satisfactory approximation, although see later for further discussion. Denoting the forcing function for individual *i* by BG_*i*_(t), we then have


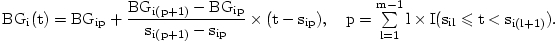


### 3.3. Calibration

**Figure 1:**
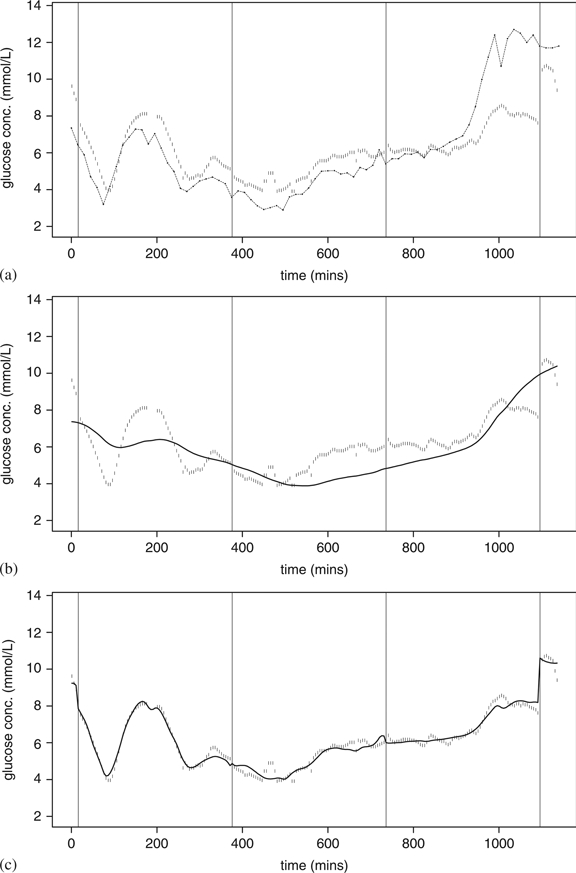
Observed data and posterior median model-predicted concentrations for individual ‘10’ plotted against time since beginning of study: (a) observed BG (−· − ) and SG (|) concentrations; (b) observed SG concentrations (|) and model-predicted values NIG_10*j*_ (—) from ‘basic’ population model; and (c) observed SG concentrations (|) and model-predicted values CIG_10*j*_ (—) from ‘calibrated’ population model.

As demonstrated in Figure 1(b), the simple model above can perform poorly. With some careful thought, however, as to the nature of the underlying calibration mechanism, we can do much better. We stress, though, that the details of calibration are actually unknown to us—implementation details of the calibration procedure are proprietary and comprise guarded know-how by the respective CGM-system manufacturers to retain competitive advantage. In what follows, we make basic assumptions about how the process *might* work in order to construct a reasonable model. We first assume that SG is given by *A* × *I*_*m*_ + *D*, where *A* and *D* are unknown constants and *I*_*m*_ is the electrical current measured by the sensor, which we assume is subject to some error, δ, such that *I*_*m*_ = *I* + δ, where *I* is the true current. We also assume that the true current *I* is related to interstitial glucose IG through *I* = IG/ S + I_*B*_, where *S* denotes *current sensitivity* and *I*_*B*_ represents a baseline current that is present even in the absence of IG. Hence,


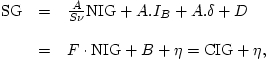


where CIG = F·NIG + B denotes ‘calibrated interstitial glucose’. Note that *A*, *S*, ν, *I*_*B*_, δ, and *D* are all unknown and so only *F* = *A*/*S*ν, *B* = *A*·*I*_*B*_ + *D*, and η = *A*·δ can feasibly be identified. Note also that while *S*, ν, *I*_*B*_, and Var(δ) might all reasonably be assumed constant, both *A* and *D* change every time the sensor is calibrated, leading to new values for *F*, *B*, and Var(η) anyway.

Let ψ_*ik*_ denote the *k*th calibration time for individual *i*(*k* = 1, …, *K* = 4). For convenience, we also define ψ_*i*0_ = *t*_*i*1_ and ψ_*i*(*K* + 1)_>*t*_*in*_. We can then write down the calibration period to which each SG_*ij*_ belongs as


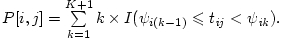


(In this paper, the calibration times are assumed known, but, in general, we may wish to acknowledge some uncertainty regarding their values.) A more realistic model for SG observations is then


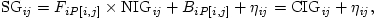
(6)

where *B*_*ik*_ and *F*_*ik*_(*k* = 1, …, *K* + 1) are unknown, individual, and calibration-period-specific parameters referred to as the calibration shift and calibration scale-factor, respectively.

We consider two different models for the residuals η_*ij*_. Assuming that the only source of ‘error’ is the measured current, then a homoscedasticity assumption leads to 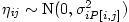, *i* = 1, …, *N*, *j* = 1, …, *n*, whereas an AR model gives


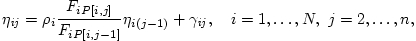
(7)

with 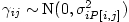, *i* = 1, …, *N*, *j* = 1, …, *n*. Here ρ_*i*_ is an unknown individual-specific parameter controlling the degree of autocorrelation among the residuals. Note that in order to obtain the *F*_*iP*[*i, j*]_/*F*_*iP*[*i, j-*1]_term, which is equal to one except at the calibration times, when it ‘adjusts’ the AR process, we make the assumption that current sensitivity, *S*, and νdo not change over time for a given individual. We cannot specify the autoregressive model, as given by (6)–(7), in BUGS, however, since a logical relationship for the response variable is not allowed. One way to get around this is to assume that each individual's SG-series arises from a CAR distribution 42, 43. A more flexible and intuitive approach, though, is to reexpress (6)–(7) as


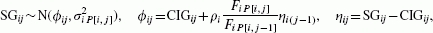
 for *j* = 2, …, *n*.

To define η_*i*1_, we could simply specify η_*i*1_ = SG_*i*1_ − CIG_*i*1_. However, note that the AR model does not penalize large ηs, and so unless we control their size, through an informative prior on η_*i*1_, say, they can become large and force the underlying ‘model fit’ {CIG_*ij*_, j = 1, …, n} away from the observed data, leading to implausible parameter estimates (see later for discussion). Note, though, that as the initial condition for the differential equation is unknown, we are free to choose the time to which it relates. If we choose the time of the first sensor reading *t*_*i*1_, then we may express the initial condition deterministically:


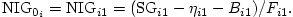


**Figure 2:**
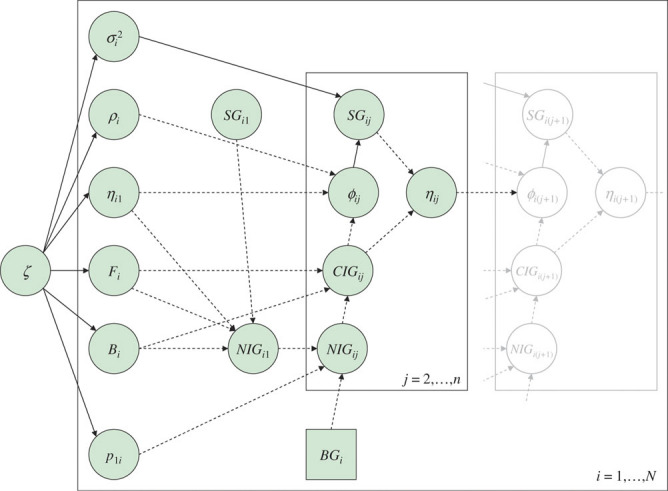
Directed acyclic graph (DAG) corresponding to ‘calibrated’ model with AR(1) process for the residual errors. For simplicity, the case in which there is only one calibration period for each individual is depicted. Each variable in the statistical model corresponds to a *node* and links between nodes show direct dependence. The graph is *directed* because each link is an arrow; it is *acyclic* because by following the arrows it is not possible to return to a node after leaving it. Square nodes denote known constants whereas circular nodes represent either deterministic relationships (i.e. functions) or stochastic quantities, i.e. quantities that require a distributional assumption. Stochastic dependence and functional dependence are denoted by solid and dashed arrows, respectively. Repetitive structures, such as the ‘loop’ from *i* = 1 to *i* = *N*, are represented by ‘plates’, which are nested if the model is hierarchical. The ‘plate’ in light-type on the right-hand side is shown to indicate the nature of dependence between successive observations. Nodes ζ and BG_*i*_ denote the entire set of population parameters, and the set of observed blood glucose concentrations for individual *i*, {BG_*il*_, l = 1, …, m}, respectively.

Hence, specifying a prior for η_*i*1_ means that there is no need to model the initial conditions. Figure 2 shows a graphical representation of the full model in the case of autoregressive errors.

### 3.4. Priors

Calibration shifts and scale-factors may be correlated; in addition, scale factors must be positive whereas shifts may be negative. We therefore define 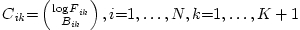, which we assume arise from a bivariate normal ‘population’ distribution. If we believe that calibration parameters reflect characteristics of the individual (and/or sensor), then we may wish to assume individual-specific means ω_*i*_ and an intra-individual covariance Ω: *C*_*ik*_∼MVN_2_(ω_*i*_, Ω). We may then wish to assume that the ω_*i*_s also arise from a bivariate normal distribution, with unknown ‘global’ mean µ and *inter*-individual covariance Σ:


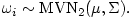
(8)

If, on the other hand, we believe that there is no correlation among calibration vectors for the same individual, then we might assume that it is the *C*_*ik*_s that are drawn from the right-hand side of (8) instead.

We assume fairly standard, vague (but proper) priors for µ, Ω, and Σ: bivariate normal and inverse-Wishart, centered at our best *a priori* guess with large variance. Throughout, the prior standard deviation specified for vague normal priors is 100 whereas Wishart priors are made as vague as possible by setting the degrees of freedom equal to the dimension, two in this case. The remaining parameters, σ_*ik*_(*i* = 1, …, *N, k* = 1, …, *K* + 1), ρ_*i*_ (where appropriate), *p*_1_*i*__, and the initial conditions NIG_0_*i*__ or initial residuals η_*i*1_(*i* = 1, …, *N*), are transformed appropriately and assumed to arise from normal population distributions. Except for the initial residuals, these population distributions have unknown means and log-standard deviations with vague normal priors. The population mean initial residual is assumed to be zero and the population standard deviation is assigned an informative uniform prior on (0, 0.5), where the upper bound ensures that initial residuals greater than one are unlikely. The transformations applied are logarithmic for the residual standard deviations, initial conditions and *p*_1_ parameters, and logistic for the ρ_*i*_ parameters (no transformation is required for the η_*i*1_s).

### 3.5. Implementation issues

Dynamic models in BUGS are ‘packaged’ in one of two ways 33. One option is to specify the differential equations using the BUGS language and pass these as arguments to a generic ordinary differential equation (ODE) solver. The alternative is to edit and compile a template module for ‘hard-wiring’ the ODE system into the software. In so doing, we create a new logical function in the BUGS language, which provides access to the numerical solution. We pass any parameters required to define the ODE system as arguments to the new function.

BUGS relies heavily on graphical modelling theory 29, 30. However, it is important to note that in graphical modelling terms, forcing functions are non-standard nodes. At first glance we might think they are logical nodes, since they are deterministic in nature. However, logical nodes are deterministic functions of other nodes in the graph: when they are defined in terms of ‘time’ they are functions of *specific* times, whereas forcing functions are defined for *all* times, in particular, they are functions of the dummy variable of integration, which, technically speaking, does not belong in the graph (Figure 2). In cases where the ODE-system is defined using the BUGS language, however, the integration-dummy is accessible within the model description, since it is required to define the differential equations. Hence, we can also use this to define forcing functions, linearly interpolating between the ‘forcing data’ via the BUGS language. One very significant advantage of hard-wiring the ODE-system, however, is that it is potentially much faster to compute. In this case, the integration-dummy is only available within the hard-wired module, and so we need to pass the forcing data, along with the times to which they relate, as parameters to the new function, and perform the interpolation within—see the Appendix.

## 4. Results

We begin by fitting the basic model given by (5), with the individual-specific parameters, *p*_1_*i*__, NIG_0_*i*__, and σ_*i*_, assumed to arise from log-normal population distributions with unknown means and standard deviations assigned vague normal and log-normal priors, respectively. We run 10 000 iterations in WinBUGS 1.4.3 31, 32 with *WBDiff* interface 33 installed. WinBUGS code for the main models considered in this paper is given in the Appendix.

Primarily, we work with ‘hard-wired’ systems of equations by writing and compiling specialized BUGS-modules, but, in this case, we also considered specifying the model entirely via the BUGS language. The former took around 5 min on a 2.13-GHz machine (when coded efficiently—see the Appendix), whereas the latter approach was substantially slower, taking ∼50 min. Point and interval estimates for the population parameters are presented in Table I, and a typical model fit is shown in Figure 1(b)—individual ‘10’ was chosen as their data best illustrate the incremental benefit of increasing the model complexity.

**Table I:** Posterior median point estimates for population parameters (mean and SD), with 95 per cent credible intervals in parentheses, from analysis of Guardian® RT SG-BG data using three different models.

	Basic model		Calibrated model		Calibrated model + AR	
			
Parameter	Pop. mean (95 per cent CI)	Pop. SD (95 per cent CI)	Pop. mean (95 per cent CI)	Pop. SD (95 per cent CI)	Pop. mean (95 per cent CI)	Pop. SD (95 per cent CI)
log *p*_1_	−3.58	0.889	−2.79	0.164	−2.82	0.166
	(−4.14, −3.03)	(0.597, 1.52)	(−2.89, −2.67)	(0.102, 0.283)	(−2.94, −2.71)	(0.0933, 0.301)
log *F*	—	—	−0.198	0.316	−0.202	0.298
			(−0.291, −0.108)	(0.258, 0.396)	(−0.289, −0.118)	(0.245, 0.370)
*B*	—	—	1.52	1.76	1.63	1.37
			(0.981, 2.06)	(1.41, 2.24)	(1.19, 2.06)	(1.08, 1.83)
log σ	−0.130	0.320	−1.42	0.615	−2.14	0.445
	(−0.329, 0.0707)	(0.216, 0.537)	(−1.60, −1.24)	(0.492, 0.782)	(−2.27, −2.01)	(0.357, 0.564)
log *NIG*_0_	2.31	0.458	2.20	0.588	—	—
	(2.01, 2.60)	(0.310, 0.750)	(1.83, 2.56)	(0.395, 0.980)		
η_.1_	—	—	—	—	0	0.374 (0.0413, 0.496)
ρ	—	—	—	—	0.8 (0.8, 0.8)	—

Visual inspection of the model fits confirms that they are generally poor. Of primary importance in this paper is our ability to predict new data, and, to this end, the basic model is clearly inadequate. We might still wonder, however, whether it provides meaningful parameter estimates. From a clinical perspective, we are interested in the time delay that exists between glucose appearing in the blood and it then showing up on the sensor. This can be seen by looking at the relative positions of BG and SG peaks and/or troughs in Figure 1(a). The time delay is represented in the model by 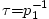. To get a rough idea of its size purely from the data, we performed a crude correlation analysis in which the SG values were lagged by 0, 5, 10 min, etc., and the correlation between BG and lagged-SG was calculated. The largest correlation coefficient (0.937) was obtained at a lag of 15 min, suggesting that a model-based estimate of the population median delay (from Table I) of exp(3.58)≈36min could be somewhat inaccurate.

To fit the ‘calibrated’ model given by (6) with 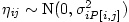, we need to choose between the available exchangeability assumptions for the calibration parameters *C*_*ik*_, *i* = 1, …, *N*, *k* = 1, …, *K* + 1. In particular, we wish to explore whether or not the calibration parameters reflect characteristics of the individual (and/or sensor), that is, whether to include individual-level means for these parameters. To address this we fit the model with and without individual means, and assess performance by looking at the posterior mean deviance, as a measure of model fit, and the Deviance Information Criterion, which penalizes the former by adding a penalty equal to the ‘effective number of parameters’ 44. We find that there is no support for individual-level means as the mean deviance is virtually the same for both models, regardless of the effective number of parameters. Hence, we proceed with a three-level, as opposed to a four-level, model.

We ran 300 000 iterations of WinBUGS for the three-level calibrated model in a little over 3.5 h. To reduce the amount of computer memory required to store the output, we retained only every fifth sample for each parameter. The final 50 000 of the resulting 60 000 samples were then used for inference. The increased run-length here is due to a high level of autocorrelation in the output for the newly introduced *C*_*ik*_ vectors. Parameter estimates for the population parameters are shown in the fourth and fifth columns of Table I. Now the population median time delay is 16.3 min, which is consistent with our crude empirical estimate. Theinter-individual variability is relatively low, corresponding to a coefficient of variation, on the time-delay scale, of around 18 per cent. This is most usefully expressed, however, in the form of a prediction interval for new individuals' time delays, which accounts for uncertainty in the population mean and variability estimates: a 95 per cent interval is given by (11.1, 23.6) min. Population medians and 95 per cent prediction intervals for the calibration shift and scale factor are 1.52 (−2.02, 5.09)mmol/ L and 0.821 (0.431, 1.55), respectively. Note that the population median residual standard deviation has reduced from exp(−0.130) = 0.878 to exp(−1.42) = 0.242, indicating a substantially better fit to the data, as illustrated in Figure 1(c).

Although the model fit is much improved, we would still like, for prediction purposes, to be able to track the considerable fluctuations away from such fits that are apparent in the data. As can be seen in Figure 1(c) the residuals are serially correlated, and so we attempt to model them via the autoregressive (AR) process (7). However, a problem arises when we run the MCMC simulation. Recall that there is no penalty for large η_*ij*_s, only their stochastic components γ_*ij*_ (see (7)) need to be small. A good fit to the data, then, can often be obtained by setting ρ_*i*_ = 1 and choosing other parameters such that the underlying ‘model fit’ {*CIG*_*ij*_, *j* = 1, …, *n*} lies a roughly constant (with respect to time) distance away from the data. Then the residuals are all similar, consistent with ρ_*i*_ = 1, and each requires only a small stochastic component. We have some control over this phenomenon in specifying an informative prior for the initial residual η_*i*1_. However, it still occurs for some individuals unless we constrain the value of ρ, and while the resulting model fits well in terms of {ϕ_*ij*_, *j* = 1, …, *n*}, the underlying CIG_*ij*_ values are often implausible.

**Figure 3:**
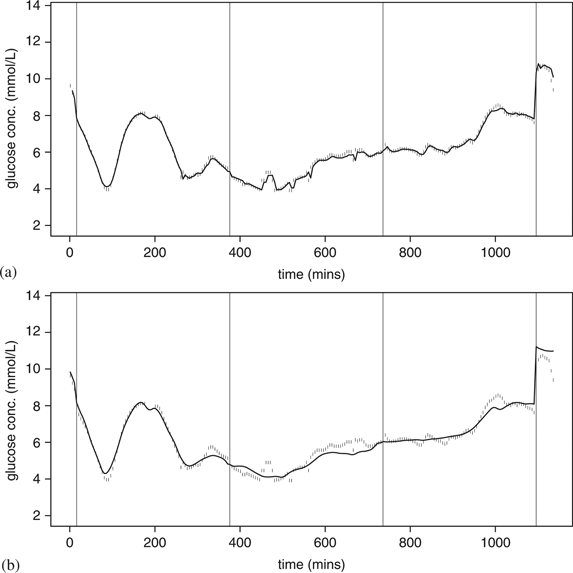
Observed SG data (|) and posterior median model-predicted concentrations for individual ‘10’ plotted against time since beginning of study: (a) ϕ_10*j*_ (—) from ‘calibrated + AR’ population model; and (b) CIG_10*j*_ (—) from ‘calibrated + AR’ population model.

**Figure 4:**
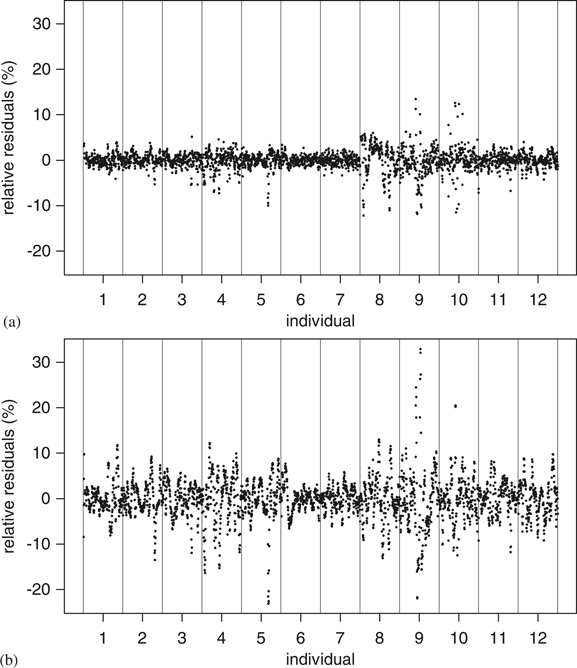
Relative residuals, 100 × (SG_*ij*_ − ϕ_*ij*_)/ϕ_*ij*_, for all 12 individuals. Residuals are plotted in time-order within each individual's zone: (a) ‘calibrated + AR’ population model; and (b) ‘calibrated’ population model.

To impose the required constraint we assume that logit(ρ_*i*_/ρ_max_), as opposed to logit(ρ_*i*_), arises from some normal population distribution (with unknown parameters), where ρ_max_ is assigned a specific value. However, now all of the individual ρ_*i*_s are estimated equal to ρ_max_, whatever value of ρ_max_ we choose. We address this by choosing the maximum value possible (in increments of 0.05) that still leads to plausible CIG-series for all individuals. Note that in so doing we find that there is no support for individual-specific ρ_*i*_s, and so we also set ρ_*i*_ = ρ ∀*i*, where logit(ρ/ρ_max_) is assigned a vague normal prior. The value chosen for ρ_max_ was 0.8, and WinBUGS was again run for 300 000 iterations, retaining only every 5th sample. This took around 11.5 h, and point and interval estimates for the population parameters from the final 50 000 samples are presented in Table I. These are in good agreement with results from the previous model (without the AR process). The calibration shift *B* seems a little higher with less variability but we would expect from these figures that the underlying CIG-series are similar to before, as illustrated in Figure 3(b) for individual 10. Note that the population median residual standard deviation is now around half its previous value, at 0.118. This corresponds to the stochastic component of the residuals γ_*ij*_ = SG_*ij*_ − ϕ_*ij*_, indicating that the ϕ-series offer a substantial improvement over the CIG-series, as we would hope, and as is demonstrated in Figure 3(a) for individual 10. To demonstrate the performance of our model across all individuals we present relative residuals, 100 × (SG_*ij*_ − ϕ_*ij*_)/ϕ_*ij*_, for *i* = 1, …, *N* = 12, *j* = 1, …, *n* = 228, in Figure 4(a). (Relative residuals are chosen in preference to the γ_*ij*_s as the percentage scale, on which they are defined, is more intuitive.) Ninety five per cent of all relative residuals have magnitudes less than 4.2 per cent, whereas 80 per cent are smaller than 2.1 per cent. The median size is 0.93 per cent. Note that individual 10 is fairly representative; if anything, he/she is one of the less well-fitted individuals. To further demonstrate the impact of the AR component in our model, we also present, in Figure 4(b), relative residuals for the case in which it is not present. Here the median relative residual size is 2.3 per cent, whereas 95 per cent of residuals have sizes below 8.9 per cent, and 80 per cent have sizes below 4.9 per cent. It is of interest to examine whether residuals corresponding to hypoglycaemic (≤3.9mmol/ L), euglycaemic (>3.9mmol/ L, ≤10mmol/ L), and hyperglycaemic (>10mmol/ L) glucose ranges are similar or not. Some small differences are apparent, with median residual sizes (inter-quartile intervals in parentheses) for the three groups given by 0.241 (0.144, 0.279) mmol/L, 0.0650 (0.0280, 0.124) mmol/L, and 0.100 (0.0400, 0.190) mmol/L, respectively. (Note that the hypoglycaemic figures are based on only 15 residuals.) Visual inspection of various plots (not shown), including histograms of residual size for each group, and plots of all residuals versus the corresponding glucose values, reveal only small trends, however, and suggest that modifying the model for the residual variance would be of little practical benefit.

Finally, we wish to acknowledge some uncertainty regarding our choice of upper bound ρ_max_. Choosing a value for ρ_max_, it seems, is tantamount to fixing ρ at that value. We would like to acknowledge that ρ could lie between 0.75, which gives inferior model fits {ϕ_*ij*_, *j* = 1, …, *n*}, and 0.85, which leads to implausible CIG-series. But there is no point trying to acknowledge this uncertainty via a prior distribution, since we know that the posterior will be concentrated on the upper boundary (we may as well set ρ = 0.85). Instead we specify ρ as a ‘distributional constant’—a fixed *distribution* as opposed to a fixed value (see 45, for example). We specify ρ∼Unif(0.75, 0.85) but we prevent learning about ρ from the likelihood—in graphical modelling terms, ρ acts as a parent of {SG_*ij*_, i = 1, …, N, j = 1, …, n} but the SG_*ij*_s are not considered to be children of ρ. WinBUGS code for ‘cutting feedback’ from the likelihood in this way is presented in the Appendix, and further comments on the use of such techniques are given in the discussion below. Results from this model give point estimates for population parameters identical to those obtained with ρ_max_ = 0.8, modulo Monte Carlo error, except for a very slight difference in the population median σ, which is still given by 0.118 to three significant figures.

## 5. Discussion

We have characterized the relationship between BG and SG, in children and adolescents, for the Guardian® RT CGM system. Various hierarchical models have been explored to determine the most appropriate model, which assumes that calibration parameters for the same individual/sensor are not correlated. Our model can be viewed as a hierarchical, nonlinear regression, where the regression function is given by numerically solving a differential equation with accompanying initial condition, forcing function, and unknown parameter. While the forcing function is not naturally accommodated within the BUGS software, due to it representing a new class of graphical node, we have illustrated how it can be incorporated.

Even without modelling autocorrelation among the residuals our model fits remarkably well, given its simplicity. However, there are clear, unexplained fluctuations away from such fits, which we have accounted for by simultaneously fitting the nonlinear regression and an AR(1) process for the residuals.§ While this has presented several practical challenges, we emphasize that the resulting model fits are very satisfactory (the majority of residuals—nearly 80 per cent—correspond to percentage differences between data and model fit of less than 2 per cent), and predictions from the model faithfully reproduce features present in the observed data. *Sudden* deviations away from the fitted curve, which are more prominent with other CGM systems, might be better handled by assuming heavier tailed, *t*-distributed stochastic residuals (γ_*ij*_) in the AR process, but we have not explored this yet. Note that Breton and Kovatchev 28 find the (unbounded) Johnson family of distributions 46 useful for the FreeStyle Navigator™ system. Another possible modification to the model for the residuals is to allow for different behaviour in hypoglycaemic, euglycaemic, and hyperglycaemic glucose ranges. However, any differences that could reliably be ascertained from our analyses were small, and it was thought that adapting the model would be of little practical benefit, especially considering that increasing the complexity of the residual model may exacerbate the apparent identifiability problem.

The motivation behind this work is to accelerate artificial pancreas development by providing a means of simulating large quantities of *realistic* sensor data, which can be fed into the control algorithm to test its response 17. To this end, it is important to account for all sources of variability in glucose-sensor data. In particular, we have estimated the population distribution of regression parameters, including their means/medians, their inter-and intra-individual variabilities, and any correlations that may exist between parameters. This allows us to generate ‘virtual patients’ by simulating realistic parameter sets from the population distribution. In addition, the Bayesian nature of our model enables full acknowledgement of the uncertainty associated with each population parameter estimate when simulating the virtual patients. Each virtual patient's parameters can be input, along with observed or simulated BG data, into the nonlinear regression function to derive CIG-values. However, the regression function is parsimonious and simply adding Gaussian white noise to this is insufficient for the purposes of generating realistic sensor data. Hence, characterizing the residuals has been a vital step in our analyses. The resulting parameter estimates can be used, again fully acknowledging their uncertainty, to simulate AR processes to be added to the derived CIG-series.

It is somewhat disappointing that we have had to constrain the degree of autocorrelation ρ in the AR process and that the posterior distribution for ρ is then always concentrated on the artificial boundary. This is mainly due, we think, to an inability to encapsulate within our prior distribution our ‘common sense’ knowledge as to the relationship between the observed data and the model-predicted CIG-series. Basically, we believe that the CIG-series should fit the data reasonably well and that the AR process should then account for mild fluctuations around that fit. But the unconstrained posterior is located near ρ = 1 with CIG-series that often lie, implausibly, some (roughly) constant distance from the data. The resulting individual-level parameters, *B*, *F*, and *p*_1_ are clearly inappropriate for the given individual, but do not necessarily have outlying values in terms of the population distribution of those parameters. Hence, it is not possible to circumvent the problem by constraining *B*, *F*, and *p*_1_. We have to remember that our model represents a gross simplification of the underlying process. To put too much emphasis on fitting the data when the model is known to be ‘wrong’ would be a mistake, in our opinion—the model is designed to provide meaningful parameter estimates and reasonably realistic predictions, which we believe it achieves.

One approach to avoiding the confounding problem would be to perform the analysis in two stages. We could first fit the calibrated model without the AR process and use this for inference on the parameters. We could then apply an autoregressive model to the residuals in order to characterize any fluctuations away from the deterministic model. However, this would ignore uncertainty in the model fit and prevent the model fit being adjusted, even slightly, to accommodate the different error structure. Hence, our efforts to fit nonlinear regression and AR models simultaneously. However, fixing ρ_max_ is tantamount to fixing ρ and we would prefer to acknowledge some uncertainty regarding the latter. We achieve this by specifying ρ as a ‘distributional constant’, as opposed to a fixed value, by placing a valve in the graphical model that allows information to flow from prior to likelihood but not vice versa. This allows us to be uncertain about ρ without the model fit being ‘tweaked’ inappropriately. Point estimates provided by this approach are identical to those obtained with ρ_max_ = 0.8, but we would prefer to use the former for prediction as the level of uncertainty would be more realistic. Cutting the feedback from one or more sources of likelihood in such a way is growing in popularity—see, for example,^45^, ^47^–^54^. The motives vary, but it is typically used for ‘multiply imputing’ missing data or combining different sub-models that might otherwise be somewhat inconsistent, due to misspecification, say.

Other ways to cut feedback in the model begin with duplicating the SG data. A homoscedastic model could be specified for the first set of data and the same *B*_*ik*_, *F*_*ik*_, and *p*_1_*i*__ parameters could then be used to define the CIG_*ij*_s needed for fitting an AR model to the second set of data. Without appropriate valves/cuts in the graph, this would lead to excessive precision due to using the data twice. If we cut the feedback from the second set of data to the *B*_*ik*_, *F*_*ik*_, and *p*_1_*i*__ parameters, however, then we are guaranteed plausible CIG-series regardless of what happens to ρ. Note that ρ can then be unconstrained—the posterior median and 95 per cent credible interval from our analyses are 0.925 and (0.908, 0.941), respectively (results for other parameters are the same as in columns 4 and 5 of Table I). This is a Bayesian analogue of the two-stage approach described above where uncertainty in the model fit is now acknowledged. Further research is required to address whether or not this approach is preferable to specifying ρ as a distributional constant. Another option would be to also create duplicate *B*_*ik*_, *F*_*ik*_, and *p*_1_*i*__ parameters, a set for each model fit, and to cut feedback from the AR set to the common population parameters (see 45, for example). This does not help for our data, however, as the parameters for the AR fit become implausible with unconstrained ρ.

One area that we have not considered in this paper is uncertainty in the forcing function. Here we have simply linearly interpolated between a series of observed values, but it is natural to think that those observations might be subject to some error, resulting in a somewhat jagged forcing function. This is likely at odds with our prior beliefs as we might expect a biological process to be largely smooth. Moreover, if there is noise in the time-series then, presumably, we would rather use the underlying ‘true’ values. Hence, we may wish to specify a separate sub-model for the BG data. This is the subject of ongoing research and will form the basis of a future report. Another methodological issue is the numerical stability of ODE solving algorithms, which can be sensitive to the values of the input parameters, in some more complex settings precluding the use of vague prior distributions, say. The works of Ramsay *et al.* 55 and Campbell 56offer an alternative approach that circumvents this problem. However, it is, as yet, unclear to us how this might be implemented in a flexible modelling framework such as BUGS or JAGS 57. More robust solving algorithms for BUGS are currently under investigation.

## Footnotes

‡ The numerical differential equation solver needs to be able to evaluate BG(*t*) at any time, not just those times at which BG has been observed.

§ The order of the AR process was chosen by fitting individual sets of posterior mean residuals using the ‘ar’ package in *R*.

## Appendix A: WinBUGS/WBDiff code

WinBUGS code for the calibrated model (without AR errors) is presented below. Most of the code is self-explanatory but some notes, pertaining to the line numbers given in the right-hand margin, are provided below for clarity.

- Line 4: The P[, ] variable, representing the calibration period to which each observation belongs, is fixed as the calibration times are assumed known. Hence, it can be defined in the data set as opposed to being calculated in the BUGS code.
- Lines 7–8: Normal distributions in BUGS are parameterized in terms of mean and precision (1/ variance). Hence, .
- Line 10: glucose(.) is the name given to our new ‘hard-wired’ function that specifies and solves the differential equation (4) at a specified grid of time-points. It is a vector-valued function of five arguments: (i) the initial condition for individual *i*, NIG_0_*i*__, denoted init[i]; (ii) the vector of times at which the solution is to be evaluated; (iii) a set of parameters required to fully specify the differential equation—these are defined on lines 11–15 (see below); (iv) the time to which the initial condition applies; and (v) the numerical tolerance to be used by the solving algorithm in determining whether or not the solution is sufficiently accurate—a value of 10^−6^ is typical. Pseudo-code for the hard-wired function is given in Appendix B.
- Lines 11–15: In order that our new, hard-wired component can evaluate the forcing function, we must supply it with the forcing data, and the times to which they relate. More generally, we may also need to supply the *number* of forcing data but here this is the same for all individuals, and so this information can be hard-wired instead of being passed as a parameter. The only other parameter required to fully specify the differential equation is *p*_1_*i*__.
- Line 19 onwards: Throughout, mean.x and prec.x denote the unknown population mean and precision of appropriately transformed x.
- Lines 47–48: zero[], prior.prec.C[, ], and prior.mat.C[, ] are specified in the data set: they are given by a two-dimensional vector of zeros, 0.0001 × *I*_2_, and *I*_2_, respectively, where *I*_2_ denotes the 2 × 2 identity matrix.

The above model is adapted to incorporate autoregressive errors (with a common autocorrelation parameter ρ for all individuals) by first making the j-loop on line 6 run from 2 to n rather than 1 to n and by replacing CIG[i, j] on line 7 with phi[i, j]. The following code is then inserted into that j-loop.

In addition, lines 18–19 above are replaced by

and lines 32–35 are replaced by the blocks of code labelled A and B as follows:

If we wish to specify rho as a ‘distributional constant’ instead, then we may replace block A with, for example,

Here the cut(.) function makes a ‘copy’ of the argument rho.dc. This always has the same value as the argument, and so uncertainty is propagated into the graph, but the ‘cut’ acts as a valve in the graph, preventing information from flowing through rho in the other direction (back to rho.dc). Hence, feedback from the likelihood is prevented and no learning about rho.dc can occur.

## Appendix B: Pseudo-code for hard-wired differential equation with forcing function

Here we present pseudo-code for evaluating the differential equation(s) in our ‘hard-wired’ module. (The reader is referred to the WBDiff documentation 33 for more general information.) The input parameters, including the forcing data, are available via a vector named theta and we are required to evaluate the equation(s) at some arbitrary time t. Note that the elements of theta are arranged as defined by the BUGS code in Appendix A, i.e. *m* forcing times, followed by *m* forcing data, followed by *p*_1_. Note also that the current solution at time t is also made available by the solving algorithm—this is denoted by NIG in the pseudo-code below. The main difficulty is in finding which elements of the forcing data are relevant to time t, i.e. which successive pair of observations lie either side of t, so that we can interpolate between them. We should be aware that the code will be called many, many times in solving the equation(s), and so it may be prudent to think about efficiency. Here we realize that each time the code is called, there is a very good chance that the ‘forcing interval’ will be the same as that most recently used or the subsequent interval. Hence, each time we evaluate the equation(s), we store, in a global variable named prev, the index of theta corresponding to the end of the forcing interval, so that we can refer to it next time.
